# Clinical Outcomes of the Deleterious Effects of Aluminum on Neuro-Cognition, Inflammation, and Health: A Review

**DOI:** 10.3390/nu15092221

**Published:** 2023-05-08

**Authors:** Guilherme Renke, Vanessa Borges Pinheiro Almeida, Everton Almeida Souza, Suzana Lessa, Raila Linhares Teixeira, Leticia Rocha, Pamela Lopes Sousa, Bernardo Starling-Soares

**Affiliations:** 1 Clementino Fraga Filho University Hospital, Federal University of Rio de Janeiro, Rio de Janeiro 21941-617, Brazil; 2 Nutrindo Ideais Performance and Nutrition Research Center, Rio de Janeiro 22411-040, Brazil; 3 Extreme Sports Nutrition Institute-INEE, Belo Horizonte 30310-370, Brazil

**Keywords:** human health, aluminum, toxicity, deleterious effects, toxicosis, antacids, oxidative stress

## Abstract

**Introduction:** In the scenario of metal toxicity, aluminum (Al) stands out as a ubiquitous type of metal that can be combined with other elements and form different compounds. Al is widely used daily as an adjuvant in vaccines, antacids, food additives (as components of AI-containing food additives), skin care products, cosmetics, and kitchenware, and can be an element or contaminant present in our daily life. **Objective:** To present a review of the main deleterious effects of Al on human health. **Methods:** The search was carried out from September 2022 to February 2023 in the Scopus, PubMed, Science Direct, Scielo, and Google Scholar databases, using scientific articles from 2012 to 2023. The quality of the studies was based on the GRADE instrument, and the risk of bias was analyzed according to the Cochrane instrument. **Results and Conclusions:** A total of 115 files were search returned. Further, 95 articles were evaluated, and 44 were included in this review. Based on the results, measuring Al’s relevance to health is essential in medicine. Several studies have demonstrated clinical outcomes and metabolic alterations with Al exposure. The tolerable weekly intake established by the European Food Safety Authority (EFSA) of 1 mg Al/kg body weight can be achieved through dietary exposure alone. Proven neurotoxicity in humans is the critical adverse effect of Al. A carcinogenic effect of Al has not been proven so far. Preventive medicine advocates that exposure to Al should be kept as low as possible. Chelating agents, such as calcium disodium ethylene diamine tetraacetic acid and deferoxamine, are options for acute poisoning, and monomethysilanetriol supplementation may be a long-term strategy with chelation potential. Further studies are needed to assess the impacts of Al on human health.

## 1. Introduction

Aluminum (Al) is the third most presented element in the Earth’s crust (just after oxygen and silicon—Al is the most frequent metal). It is typically found as a relatively insoluble aluminosilicate, which comprises 8% of the weight of the earth’s crust [1,2]. In the scenario of metal toxicity, Al stands out as a ubiquitous metal that combines with other elements to form different compounds and can also be found in ionic form with greater reactivity to human tissues. Al is widely used in daily life as an adjuvant in vaccines, antacids, food additives (as a component of AI-containing food additives), skin care products, cosmetics, and kitchenware, and can be as elements or contaminants present in many foods, including infant formula, milk products, juices, wines, seafood, tea, and vegetables. It also appears in drinking water due to the water treatment or naturally coming from weathered rocks and soils [3]. An additional increase in the presence of Al in our daily life came from the development of Al beverage cans and packaged foods in the 1970s [1].

Additionally, Al is being widely generated and used in industries (such as mining, smelting, and welding), and workers are particularly exposed to its particles. Occupational exposure to Al can increase the risk of hypertension and cognitive impairment; the hypertension effect may mediate the cognitive impairment caused [4]. Humans ingest Al through the respiratory, digestive, and skin systems [5,6,7,8,9,10]. Al can affect our health, significantly harming the central nervous system. Thus, Al can cause crucial cognitive impairment, Alzheimer’s disease (AD), and other neurodegenerative disorders [4].

Still, in this context, a question that arises from a scientific perspective is: how high is the risk of adverse health effects from sole exposure to Al [4]? It has long been established in medical applications as, for example, an adjuvant in vaccines and as an agent against pathological hyperhidrosis with studies presenting a low profile of side effects [11,12,13], but, on the other hand, there is discussion regarding if Al in deodorants can trigger breast cancer [14], corroborating the recent increase in studies presenting its neurotoxic and potential carcinogenic effect [15]. Statistical effects in the direction of “protection” carried by calcium higher than 75 mg/L and weak interaction between Al and pH in drinking water have been observed [16].

According to the preventive medicine principle of minimization, exposure to foreign potentially toxic substances should always be as low as possible. However, Al is very commonly found in the blood and urine of humans. Suppose a foreign substance exceeds its reference value (the 95th percentile value). In that case, it is questioned from a medical point of view: whether and at what concentration of the substance would it have a substantial danger to human health [17]?

In this sense, since the Second World War, several factors have contributed to a significant increase in human exposure to Al, with the tolerable intake of Al being exceeded by a significant part of the world’s population, especially in children, a group that is more vulnerable to the potentially toxic effects of Al pollutants than other groups. Faced with an oral influx of Al, the intestinal tract is an essential shield for the human body, as 38% of the ingested Al accumulates in its mucosa. Although poorly documented, oral exposure to Al under conditions relevant to humans appears harmful to intestinal homeostasis. Al ingestion can affect permeability regulation, gut microbiota, and immune function [1].

Therefore, the present study aims to review the main deleterious effects of Al on human health and introduce the physiological effects of Al antioxidants and potential chelant substances.

## 2. Methods

### 2.1. Study Design

The present study followed a concise review model, presenting the most discussed and impactful topics in the area. The most obvious highlight was for systematic review articles or meta-analyses of randomized clinical trials. According to the GRADE instrument, the low quality of evidence was attributed to case reports, brief communications, and editorials.

### 2.2. Search Strategy and Search Sources

The literature search was carried out from September 2022 to February 2023. It was obtained based on Scopus, PubMed, Science Direct, Scielo, and Google Scholar results, using scientific articles from 2012 to 2023, using the descriptors (MeSH Terms): “Human health. Aluminum. Toxicity. Deleterious effects”, and using the Booleans “and” between the MeSH terms and “or” between the historical findings.

## 3. Results and Discussion

### 3.1. Summary of Findings

**Figure 1:**
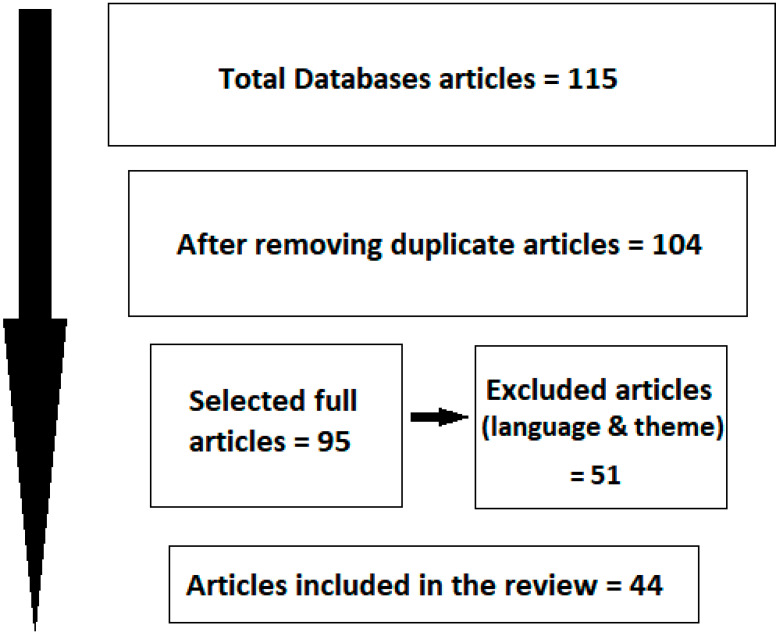
Studies selection.

As a result of the literature review, 115 files derived from the research were evaluated and subjected to eligibility analysis, and 44 of the final 95 studies were selected for this review. Exclusion criteria: papers in languages other than English and/or without health focus were excluded. The studies described presented medium to high quality (Figure 1), considering, in the first instance, the level of scientific evidence of the studies in types of study, such as meta-analysis, consensus, randomized clinical trial, prospective, and observational. The biases did not compromise the scientific basis of the studies.

### 3.2. Toxicity—Inflammation and Associated Impacts

As postulated more than 30 years ago in *The Lancet*: the move to recommend a limitation of human exposure to Al is a public policy action, and they suggested reducing Al concentrations in drinking water to 50 mg/L in the short term and 10 pg/L in the long term; no more than 3 mg total Al should be ingested daily [2]. In 2012, the French Food Safety Agency (AFSSA) used a representative market basket to determine the exposure to trace elements, including Al, coming from ordinary foods. They found that the French population’s mean exposure to Al in food is estimated to be 40.3 mg/kg bw/day in adults and 62.2 mg/kg bw/day in children [18]. This ingestion would be at the edge of the 3 mg postulated as a target. However, if we add Al from utensils and packaging (from westernized diet products), which ranges between 2 and 4 mg of AI/day, we would extrapolate the wares in different meat extracts and milk [19].

In addition, using Al salts in vaccine adjuvants to increase efficacy is one of the main reasons for this lack of confidence. Direct Al toxicity is often presented. It has long been known that direct Al toxicity, especially with occupational exposure, is associated with characteristic clinical manifestations and increased blood Al levels [20].

Thus, excessive amounts of metals such as Al are released into the environment due to the aggressive pace of human activities. Thus, chronic exposure to various metals, such as AI, is increasingly common, which has become increasingly common in our society and is a significant environmental risk factor. This metal may affect brain physiology, immunity, and its roles in accumulating toxic AD protein species (i.e., β-amyloid and tau) [21].

An interesting relationship between Al and brain damage is related to microglial hyperactivation [20,21,22]. A large scientific study showed that microglia are crucial in preventing injury and brain damage after trauma related to tumor formation and invading microorganisms. Indeed, microglia in the central nervous system are critical to the defense of brain tissue. Despite this, excessive activation of these cells can result in deleterious effects through complement activation and induction of an adaptive immune reaction and three Fcγ receptors. Intercellular adhesion molecule-1 (ICAM-1) and CD3 molecules are the substances that contribute to this adverse reaction. Thus, an autoimmune inflammatory reaction in the brain may occur earlier in glial cells before cytokine release. Round microglial cells among the pyramidal cells of the hippocampus with increased expression of CD32+ (FcγIIa) and close to the Al injection site were detected immunohistochemically. They indicated microglial activation at the Al lesion site. ICAM-1+ immunoreactivity increased significantly in the hippocampus and choroid plexus after Al injection, indicating increased CD3ξ+ expression and glial cell inflammation in the hippocampus and non-MHC-restricted T cytotoxicity. Microglia may play a phagocytic role at the site of Al-induced excitotoxicity in the brain through the CD32+ (FcγIIa receptor) expression pattern near the Al injection site. A neurotoxic autoimmune reaction initiated by microglial hyperactivation in the injured brain tissue is caused by the prominent expression of CD3ξ+ immunoreactive cells with ICAM-1+ clusters in the choroid plexus [22].

Additionally, aluminum phosphide (AlP) can induce oxidative stress, one of the most critical mechanisms of its toxicity, increasing malondialdehyde (MDA) levels and reducing total plasma antioxidant capacity [23].

### 3.3. Neurotoxicity and Cancer

The prevalence of acute Al toxicity is low, and no acute effects due to dietary exposure to Al were observed in the general population. Despite this, it is well known that Al foil is not recommended for some types of food preparation due to Al migration [24]. A recent study focused on developing an analytical method to study the presence of toxic substances from FCMs (food contact materials) in baking paper and Al foil. The study showed greater exposure of consumers to the contaminants, consuming a simulated aqueous-based acid food in both types of FCMs [25].

AI has a high affinity for proteins and is capable of cross-linking. In contrast to other ubiquitously occurring metals, such as zinc, iron, and manganese, Al’s physiological or metabolic role in the human body has yet to be discovered. In addition, relevant Al intoxication symptoms were observed in dialysis patients associated with neurotoxic effects. Indeed, it was identified that Al was the causative agent; patients had elevated serum and brain levels of Al. Affected patients experience memory impairment, dementia, and disorientation. The probable cause of these effects is Al’s slow removal and washout from brain tissue; for this reason, Al directly affects brain functioning (Table 1) [10,11].

In addition to binding to negatively charged membrane structures in neurons and favoring oxidative stress, Al can alter neuronal plasticity through changes in hippocampal calcium signaling pathways, which are also crucial for memory. Al still affects the synthesis of the neurotransmitter acetylcholine because cholinergic neurons are very susceptible to Al neurotoxicity. The last two neurobiological effects are also relevant in the supposed association between Al and AD [13]. Studies have shown that chronic Al exposure is associated with the occurrence and development of several diseases, including neurodegenerative disorders (AD, Parkinson’s disease (PD), Huntington’s disease, etc.) [24,25,26,27,28,29,30]. In a recent study, Zhu and colleagues showed that high IL-1β protein levels were detected in AI-treated mouse cerebral cortex and microglia-type BV2 cells, validating that Al could have a neuroinflammatory role. This is possibly due to AI-induced excessive activation of microglia, releasing more inflammatory mediators to injured neighboring neurons and further activating microglia and other nerve cells, exacerbating neuroinflammation, which could lead to neurodegenerative effects. Extracellular ATP (Eatp) can trigger the P2X7 receptor, leading to an inflammatory response. Al exposure could activate microglia and increase Eatp amount, activating the membrane P2X7 receptor and the nuclear factor HIF-1α, which collectively regulate the NOD-like receptor family 3 (NLRP3) inflammasome complex and synthesize inflammatory factor IL-1β, promoting an inflammatory response that may lead to neuronal damage [26].

Furthermore, detrimental changes in neuropsychological tests (concerning concentration, learning, and memory) were observed after the occupational exposure of workers, in which concentrations of approximately 100 µg of Al per gram of creatinine and about 13 µg/L of Al in plasma were used, compared to non-exposed workers. A study from Klotz and colleagues (2017) presented the well-documented adverse effects of Al on health and peak levels in humans [17]. Occupational exposure easily exceeds reference values for maximum internal Al load (<5 μg/L in serum and <15 μg/L in urine). In urine, 50 μg of Al per gram of creatinine is the maximum biological value considered for occupational exposure. Al concentrations greater than 100 μg/g of creatinine in the urine have been found in workers in the Al industry, especially welders. In them, a significant decline in performance in neuropsychological tests (memory, learning, and attention) was observed. Despite this, the presence of dementia associated with encephalopathy was not observed. However, in AD patients, an elevated amount of Al was found in the brain tissue. It is still unclear whether this is the disease’s cause or effect. Kilburn of the Environmental Sciences laboratory at the University of Southern California presented that the most common AI-attributable neurobehavioral side effects are tremors, impaired balance, decreased recall memory, and slower cognitive functions [30].

In addition, in patients with chronic kidney disease, bone disease and neurotoxicity may occur with toxic and elevated exposure to Al. Patients with stage 5 chronic kidney disease exposed long term to Al developed Al neurotoxicity at higher concentrations than those with Al bone disease or asymptomatic Al overload. However, acute exposure to intravesical Al causes neurotoxicity even at lower Al concentrations [27]. As Al accumulates in the bone of patients with renal failure, attacking about 44 percent of the patients treated with long-term dialysis [31], deferoxamine is presented as a beneficial treatment for AI-induced dialysis side effects, acting as a chelating agent. The study analyzes if the long-term intermittent infusion of deferoxamine works to clear Al deposition from bone, demonstrating that the injection of deferoxamine removed Al from the bone of patients undergoing long-term dialysis, reducing bone pain and improving physical activity after 2–4 weeks of treatment [32].

Regarding cancer risk, whether using Al-containing antiperspirants can cause breast cancer is still controversial. An increase in tumors in the upper outer quadrants of the breast has been observed in recent years. However, this breast area is also the richest in glandular tissue. As proof of this, high levels of Al have been obtained from patients with breast cancer (liquid aspirated from the nipple). Al concentrations were higher in the outer quadrants compared to the inner quadrants [14].

### 3.4. Absorption

Based on the studies selected in this review, it was shown that the mean daily dietary intake of Al is 1.6–13 mg (0.2–1.5 mg/kg BW/week), and only about 0.1% of Al ingested orally is absorbed by the gastrointestinal tract and becomes bioavailable [10]. The tolerable weekly intake (TWI) established by the European Food Safety Authority (EFSA) of 1 mg Al/kg body weight (BW) in a 60 kg adult is, in some individuals, already saturated or above. Relative exposure in children is the highest, up to 2.3 mg/kg BW/week. More significant amounts can be ingested (1g or more per day) by people taking antacids with Al hydroxide as the main ingredient [33]. TWI levels are designed to be preventive, and long-term values for the general population must be established [5,10].

Furthermore, exposure to Al is highly dependent on the form of exposure (Table 2). Exposure through the skin and gastrointestinal tract is minimal in humans, and there is a specific situation in which the absorption could be potentialized: stripped skin (a procedure equivalent to shaving) can absorb ten-times more Al chloralhydrate than intact skin [34]. Therefore, the TWI value is only limitedly adequate to reflect the organism’s exposure to Al. Endogenous exposure, typically determined by serum or urine Al levels, is a better way to assess Al-related neurotoxicity [6].

In addition, a clinical study evaluated AI, Zn, and Pb levels in occupationally exposed workers from northwest India and raised awareness of their toxicity and adverse outcomes. This study evaluated 120 exposed workers and 100 controls (unexposed), ranging in age from 18 to 78 years. Pb, Al, and Zn serum levels were estimated using atomic absorption spectrophotometry. The results showed significantly higher AI, Zn, and Pb levels in the exposed subjects than in the unexposed subjects. No significant differences were observed in metal levels based on age and duration of exposure. Painters had higher serum levels of Pb, while welders had higher serum levels of Al and Zn [39].

### 3.5. Diagnostic Test and Excretion

AI and its external sources may increase blood levels from contamination during processing, collection, or analysis. Patients exposed to Al poisoning present clinical findings when blood and urine samples are collected, which is a crucial point [40]. Generally, the amount of serum Al will be lower than 10 micrograms per liter (mcg/L) or 60 mcg/L in dialytic patients. Toxicity occurs at concentrations of higher than 100 mcg/L [35]. Thus, urine Al levels below 55 micrograms per gram (μg/g) of creatinine appear safe in humans. In addition, urinary Al concentrations ranging from 108 to 162 micrograms per liter (μg/L) indicate a neurological side effect threshold. Additionally, the development of neurological complications is related to a urine critical level of 100 μg/L [36]. Al nail, sweat, and hair levels can also be evaluated for poisoning [41]. In an exciting new study, the trace elements concentration in the hair of 118 young Japanese children was analyzed via ICP-MS. Girls had significantly higher levels of AI, iron, and copper (*p* = 0.000, 0.014, and 0.013, respectively) than boys [42].

At least 95% of Al is excreted through the kidney. AI’s primary elimination is through the kidneys, while bile Al excretion represents about 1–2%. Healthy subjects, under typical situations, can excrete all orally absorbed Al. In populations exposed to high levels of Al, in total parenteral nutrition, for example, Al cannot be adequately excreted, leading to the accumulation of Al. Al ultrafiltration ability is limited by protein binding and the primary reason for this accumulation. Al clearance depends on route, type, and frequency of exposure. Although most of the absorbed Al is excreted in 7 days after exposure, in some cases, it is estimated that the excretion process may last weeks, months, and even a few years [37,38].

### 3.6. Chelation Therapy

Chelating agents and antioxidants have been used experimentally to reduce Al’s deleterious effects in rats, mice, and cell models (Table 3). Employed experimentally, they ameliorate the adverse effects of Al in the brain, liver, kidneys, spleen, osteoblasts, and blood cells.

Regarding supplements, several studies have shown the benefits of substances with Al chelation properties. In humans, the supplementation of MMST (Monomethysilanetriol) has different applications, including preventing neurodegenerative disease due to decreased Al levels, increasing the body pool of silicon, and improving mineral density (BMD). Silicic acid in drinking water could also exclude Al absorption from other ingested sources [16]. To evaluate MMST in decreasing the levels of Al, Ferreira and colleagues (2018) conducted a randomized, double-blind, and controlled placebo clinical trial to compare the effects of M-OSA (maltodextrin-stabilized orthosilicic acid, M-OSA) and MMST in different aspects, including decreases in Al. The studied group comprised 51 women aged 40–60, divided into M-OSA, MMST, and placebo groups. For the supplemented groups, the dose of elemental silicon was 5 mg twice a day for five months. The analyses were conducted thrice: 0, 90, and 150 days. The results obtained in the MMST group were statistically significant in decreasing from 6.82 μg g^−1^ (T0) to 6.55 μg g^−1^ (T150) Al levels, representing a decrease of 3.9% (*p* < 0.05). Additionally, in the placebo group, it was observed that the Al level rose 3.5%. This may be explained by the fact that it is not possible to avoid Al exposition. Thus, it can be concluded that the supplementation of MMST can lead to a decrease of 53% in Al levels, as an accumulation of 4.10 μg g^−1^ (T150, placebo group) without the supplementation has been observed; with the supplementation and during the same period, rather than accumulation, a decrease in the heavy metal levels was observed [51].

MMST is a highly permeable and amphiphilic organosilicon molecule (CH3Si(OH)3) with good stability, even at concentrations above 20 mM at room temperature. Additionally, it has rapid/high intestinal absorption; no side effects or adverse effects have been reported in previous studies [52,53]. MMST is converted into biologically active orthosilicic acid after its intestinal absorption and, from there, interacts with Al. The exact mechanism of MMST’s chelating effect on Al has yet to be fully described. It is believed that orthosilicic acid forms complexes with aluminum hydroxide. The resulting aluminosilicates decrease the availability of free Al, thus preventing the occurrence of intoxication [51,52,53].

Individuals affected by Al intoxication may benefit, in the short term, from treatment with calcium ethylenediaminetetraacetic disodium acid (EDTA) or chelator combinations, such as ascorbate (vitamin C) and deferoxamine (DFO), that have the highest efficiency in removing Al, which the FDA recognizes as a well-known drug [54]. The efficacy of long-term treatment with EDTA was evaluated with safety parameters. Slow intravenous therapy with the chelating agent EDTA (2 g/10 mL diluted in 500 mL physiological saline administered in 2 h) removes Al, which is detected (using inductively coupled plasma mass spectrometry) in urine samples collected from patients over 12 h after the procedure. Protocols with EDTA can vary in the literature. In cases of Al intoxication (expressed in μg per g creatinine) who underwent EDTA chelation therapy, the protocol consisted of ten-week intravenous sessions [28]. Such therapy could be further improved through daily treatment with other oral antioxidants and supplements (Table 3).

Additionally, a randomized controlled clinical study developed by Halvaei et al., 2017, evaluated vitamin E’s effect in treating acute Al intoxication. The treatment group received vitamin E (400 mg/BD/IM). The malondialdehyde (MDA) level and total plasma antioxidant capacity were measured. The plasma level of MDA significantly decreased in the treatment group. Administration of vitamin E reduced the requirement (30% vs. 62%, *p* < 0.05) and the duration of intubation and mechanical ventilation (*p* < 0.05). Furthermore, it significantly reduced the mortality rate in the treatment group compared to the control group (15% vs. 50%, respectively, *p* < 0.05). Therefore, vitamin E and supportive care may be therapeutic in acute Al intoxication [23].

Further, some factors can interfere with the absorption and distribution of Al by our body, which, in turn, can progressively facilitate or contribute to the development of chronic exposures, triggering adverse consequences for our health. For example, it has been shown that encephalopathy can be caused by excess Al, as well as bone disease and anemia in dialysis patients. However, it remains controversial whether chronic exposure to low doses of Al can contribute to AD development, probably due to its multifactorial and variable nature [1].

Our study has some potential confounders (e.g., it is known that women perform some neurological tests faster than men so that gender groups may be analyzed separately and distinguishing the effects of Al and the different forms of Al compounds on human health) that should be discussed.

## 4. Conclusions

The critical adverse effect of endogenous Al accumulation is proven neurotoxicity, increased inflammation, and oxidative stress. Evaluating measured Al values regarding their relevance to health is essential in medicine. Preventive medicine strongly advocates that Al exposure should always be as low as possible. The established TWI of 1 mg AI/kg body weight can easily be achieved through dietary exposure alone. A carcinogenic effect of Al has not been confirmed so far. Intravenous chelating agents, such as EDTA and DFO, are options for acute poisoning. In addition, supplements, such as MMST, with chelation potential may be a long-term strategy. Further studies analyzing the effects of Al on human health are of great interest.

## Figures and Tables

**Table 1:** Aluminum exposure-associated detrimental effects.

Detrimental Effects	References
Oxidative stress and lipid peroxidation	Willhite, 2014 [13]; Zhang et al., 2022 [4]
Protein denaturation and disturbance	Igbokwe, 2019 [10]; Paul-ehrlich-institut, 2022 [11]
Enzymatic/Receptor function disturbance: stimulation or inhibition	Zhu, 2023 [26]
Amyloidogenic and anti-amyloidolytic action	Huat, 2019 [21]
Metalloestrogen action: promoting augmented proliferation and migration of breast cancer cells	IARC, 2012 [14]
Disrupts receptor function and cell membrane	Willhite, 2014 [13]
Increases osteoclastic activity and reduces osteoblastic activity, inhibiting bone formation and mineralization	Coulson, 2022 [27]
Alter gastrointestinal tract homeostasis (permeability regulation, gut microbiota, and gut immune function)	Vignal et al., 2016 [1]
Induces arterial hypertension (systolic and arterial)	Zhang et al., 2022 [4]

**Table 2:** Absorption/excretion/normal and toxic levels/diagnostic tests. Gastrointestinal tract (GIT); European Food Safety Authority (EFSA).

Parameter	Levels
Daily dietary intake of Al	1.6–13 mg (0.2–1.5 mg/kg BW/week) (Igbokwe, et al., 2019) [10]
Absorption	0.1% of the total Al orally ingested absorbed by GIT (Igbokwe, et al., 2019) [10]
Tolerable weekly intake	1 mg Al/kg body weight (EFSA) [33]
Diagnostic tests: urine blood serum	55 μg/g of creatinine (Oliveira, et al., 2021) [6] 10 mcg/L or 60 mcg/L (dialytic patients) (Oliveira, et al., 2021) [6]
Toxicity tests: urine blood serum	ranging from 100–108 to 162 mcg/L (Wechphanich & Thammarat. 2007) [35] concentrations higher than 100 mcg/L (Ogawa & Kayama. 2015) [36]
Main Excretion mechanisms: urine Bile	95–99% (Krewski, 2007) [37] 1–2% (Berthon, 2002) [38]

**Table 3:** Aluminum protection antioxidant/chelant effects. Intraperitoneally (i.p); chlorogenic acid (CGA); aluminum chloride (AlCl3); MMST (Monomethysilanetriol); disodium ethylene diamine tetra acetic acid (EDTA).

Antioxidant/Chelant	Physiological Effects
Chow supplemented with 1 mg/kg selenium as sodium selenite daily (Abubakar, et al., 2004) [43].	Treatment with a selenium-rich diet was associated with a significant reduction of Al in the liver and brain of animal models after infusion of an aqueous solution with Al at a dose of 5 mg/kg per day, five times a week for three weeks.
Injected (i.p.) CGA (2 h after Al-treatment, a single dose of 100 mg/kg) and CGA (administered to mice daily for 5 days at 30 mg/kg before Al-treatment) (Cheng, et al., 2017) [44].	CGA (5-O-caffeoylquinic acid) prevented induced oxidative damaging effects, hepatotoxicity and hematotoxicity of single injection (i.p.) of 25 mg AlCl3 in mice.
Pretreatment (30min before AlCl3) with oral melatonin (200 μg/kg body weight) (Fyiad, et al., 2007) [45].	Melatonin (200 μg/kg body weight) 30 min prior to aluminum chloride AlCl3 orally (8.5 mg/kg body weight for eight weeks) was associated with a decline in all abnormal changes observed in AlCl3 treated rats-oxidative damage and Al-toxicity induced injury to liver, kidney and brain.
Oral propolis (50 mg/kg body weight) (Al-Qayim et al., 2014) [46].	Propolis reduced the significant negative elevation of kidney function parameters in rats presented by the oral AlCl3 (50 mg /kg body weight) for 60 days.
Oral propolis (50 mg/kg body weight) (Turkez, et al., 2010) [47].	Propolis significantly modulated the hepatic toxic effects of oral AlCl3 (34 mg/kg body weight) 30 days in rats.
Oral acid malic (45 mg/kg body weight) (Al-Qayim et al., 2014) [46].	Acid malic reduced the significant negative elevation of kidney function parameters in rats presented by the oral AlCl3 (50 mg /kg body weight) for 60 days.
Oral tannic acid (50 mg/kg body weight) (Omar, et al., 2003) [48].	Tannic acid partially improves the moderate toxicity on liver, kidneys and spleen of treated rats with oral AlCl3 (50 mg /kg body weight) for 80 days.
Oral quercetin (10 mg/kg body body weight) (Sharma, et al., 2016) [49].	Quercetin attenuates neuronal death against oral Al lactate-induced (10 mg /kg body weight) neurodegeneration in rats for 12 weeks.
Cell culture added ginsenoside Rb1 (0.0145 mg/Ml) (Zhu, et al., 2016) [50].	After 24 h of culture, ginsenoside Rb1 alleviates AlCl3-induced (0.126 mg/mL) rat osteoblasts dysfunction.
**Human studies**	
Vitamin E (400 mg/BD/intra muscular) (Halvaei et al., 2017) [23].	DL-alpha tocopheryl acetate significantly decreased the plasma MDA level.
Oral monomethysilanetriol (10mg/day) (Ferreira, et al., 2018) [51].	The supplementation of MMST can lead to a decrease of 53% in aluminum levels 150 days in humans.
Intravenous EDTA (2 g/10 mL diluted in 500 mL physiological saline administered in 2 h) (Fulgenzi, 2015) [28].	EDTA removes Al in urine samples collected from patients over 12h after the procedure.

## Data Availability

Not applicable.
